# Cannabinoid products for pain management: recommendations from the São Paulo State Society of Anesthesiology

**DOI:** 10.1016/j.bjane.2024.844513

**Published:** 2024-05-11

**Authors:** Guilherme Antonio Moreira de Barros, Alexandre Mio Pos, Ângela Maria Sousa, Carla Leal Pereira, Cecília Daniele de Azevedo Nobre, Cláudia Carneiro de Araújo Palmeira, Cristina Aparecida Arrivabene Caruy, Derli Conceição Munhoz, Durval Campos Kraychete, Esthael Cristina Querido Avelar, Fernanda Bono Fukushima, João Batista Santos Garcia, João Nathanael Lima Torres, Karenthan de Abreu Rodrigues, Mariana Palladini, Olympio de Hollanda Chacon Neto, Maria José Carvalho Carmona

**Affiliations:** a Universidade Estadual Paulista (Unesp), Faculdade de Medicina de Botucatu, Botucatu, SP, Brazil; b Regenerar Clínica de Dor do Gama, Brasília, DF, Brazil; c Universidade de São Paulo (USP), Instituto do Câncer do Estado de São Paulo, São Paulo, SP, Brazil; d Unidade Brasil do Hospital São Luiz, São Paulo, SP, Brazil; e Casa de Saúde São José (Rede Santa Catarina), Rio de Janeiro, RJ, Brazil; f Universidade do Estado do Rio de Janeiro (UERJ), Rio de Janeiro, RJ, Brazil; g Universidade de São Paulo (USP), São Paulo, SP, Brazil; h Faculdade de Medicina da Universidade Estadual de Campinas (Unicamp), Campinas, SP, Brazil; i Universidade Federal da Bahia (UFBA), Salvador, BA, Brazil; j Universidade Federal de São Paulo (UNIFESP), São Paulo, SP, Brazil; k Universidade Federal do Maranhão (UFMA), São Luís, MA, Brazil; l Instituto Doutor José Frota (IJF), Fotaleza, CE, Brazil; m Centro Paulista de Dor, São Paulo, SP, Brazil; n Hospital Pro Matre Paulista, São Paulo, SP, Brazil

**Keywords:** Cannabinoids, Pain, Treatment outcome, Review

## Abstract

There is growing interest in using cannabinoids across various clinical scenarios, including pain medicine, leading to the disregard of regulatory protocols in some countries. Legislation has been implemented in Brazil, specifically in the state of São Paulo, permitting the distribution of cannabinoid products by health authorities for clinical purposes, free of charge for patients, upon professional prescription. Thus, it is imperative to assess the existing evidence regarding the efficacy and safety of these products in pain management. In light of this, the São Paulo State Society of Anesthesiology (SAESP) established a task force to conduct a narrative review on the topic using the Delphi method, requiring a minimum agreement of 60% among panelists. The study concluded that cannabinoid products could potentially serve as adjuncts in pain management but stressed the importance of judicious prescription. Nevertheless, this review advises against their use for acute pain and cancer-related pain. In other clinical scenarios, established treatments should take precedence, particularly when clinical protocols are available, such as in neuropathic pain. Only patients exhibiting poor therapeutic responses to established protocols or demonstrating intolerance to recommended management may be considered as potential candidates for cannabinoids, which should be prescribed by physicians experienced in handling these substances. Special attention should be given to individual patient characteristics and the likelihood of drug interactions.

## Introduction

There is worldwide interest in using cannabinoid products for management of several conditions. The United Nations (2020) acknowledged the medicinal potential of cannabinoids and excluded them from Annex IV of the 1961 Single Convention on Narcotic Drugs, enabling a less restrictive clinical use of cannabinoids.^1^ Some European countries have legalized the prescription of plant derivatives of *Cannabis sativa* for several conditions, including pain management, neglecting the usual regulatory processes foreseen for the commercialization of medicines.^2^

On January 31, 2023, the State of São Paulo, Brazil, approved law number 17,618 establishing the state public policy for the free distribution of cannabinoid products when prescribed by healthcare professionals. In Brazil, this marked the first regulation enabling medicines and herbal remedies containing cannabinoids to be systematically provided to patients through the Public Health System. The law contemplates the availability of Cannabidiol (CBD) based medicines of plant origin, in association with other cannabinoid substances, including Tetrahydrocannabinol (THC).^3^ As a result of the enactment of the law, a task force was established by the São Paulo State Health Department with the participation of representatives from different areas of society with an interest in the matter. The main objective of the working group is to establish protocols for supplying these products in different clinical scenarios, including pain.

Specifically concerning pain management, despite recent studies suggesting the benefits of medicinal cannabis, robust clinical evidence, such as that found in meta-analyses, is lacking. This is attributed to the high dropout rate of participants in clinical studies due to side effects, as well as the significant heterogeneity and inconsistency in the products and concentrations administered, which adversely affects the reliability of meta-analyses.^4^

It is believed that *Cannabis sativa* has been cultivated for over 12,000 years, though its medicinal use only emerged in China around 2,700 BC. In Western civilization, the earliest documented use occurred in 1839, when a pioneering study was conducted on its potential as an analgesic. More recently, particularly after the 1960s, extensive research has been undertaken on cannabinoids’ potential as analgesics and anti-inflammatories, as well as their ability to modulate the nociceptive pathway. However, conclusive evidence authorizing their routine prescription has yet to be established.^5^

Other factors pose challenges to acquiring clinical evidence on cannabinoids as analgesics, including practical obstacles to conduct research:^4^^,^^6^ 1) Existence of excessively restrictive regulatory measures; 2) Cannabis-based products available on the market are not covered by federal research funds, which makes difficult access for research purposes; 3) Published research has a predominance of private funding, which generates biases in results, making it urgent to create public policies to encourage research with these products; 4) There is a wide variety of drugs and products available for sale, hindering direct comparisons among them.

More than 538 chemical substances derived from *Cannabis sativa* are known. Of these, 100 cannabinoid products have been isolated, posing a massive challenge in obtaining scientific evidence for their clinical use.^7^ During the preparation of the present narrative review, 16 products containing isolated cannabidiol and 11 products containing extracts from the *Cannabis sativa* plant in various proportions were approved by the National Health Surveillance Agency (Anvisa) for sale in pharmacies in Brazil (Table 1).^8^ It is important to emphasize that the several routes of administration also pose a greater challenge to obtaining evidence. Some studies utilize the inhalation route for administering the products, which is potentially iatrogenic and not recommended by either the Federal Drug Administration (FDA) of the United States of America or the Brazilian Anvisa.

**Table 1:** Cannabis products registered with the National Health Surveillance Agency (Anvisa) in Brazil and available for sale in pharmacies, categorized by their active ingredients*.

Active Ingredient	Manufacturer	CBD Concentration	THC Concentration	Drops per mL
Cannabidiol	Active Pharmaceutica	20 mg.mL^−1^	<0.2%	NA
	Aura Pharma	50 mg.mL^−1^	NI	NA
	Belcher	150 mg.mL^−1^	NI	NA
	Collect	20 mg.mL^−1^	<0.2%	NA
	Ease Labs	100 mg.mL^−1^	<0.2%	30
	Farmanguinhos	200 mg.mL^−1^	NI	NA
	Greencare	23.75 mg.mL^−1^	<0.2%	30
	Mantecorp Farmasa	23.75 mg.mL^−1^	<0.2%	30
	Nunature	17.18 and 34.36 mg.mL^−1^	<0.2%	50
	Prati-Donaduzzi	200; 20 and 50 mg.mL^−1^	NI	NA
	Promediol	200 mg.mL^−1^	NI	36
	Verdemed	23.75 and 50 mg.mL^−1^	<0.2%	NA
*Cannabis sativa* extract	Cann 10 Pharma	200 mg.mL^−1^	<0.2%	37
	Cannabr	10 mg.mL^−1^	NI	NI
	Greencare	79.14 and 160.32 mg.mL^−1^	2.4 mg.mL^−1^ and 0.06 mg.mL^−1^	30
	Mantecorp Farmasa	79.14 and 160.32 mg.mL^−1^	0.08 mg.mL^−1^	30
	Promediol	200 mg.mL^−1^	<0.2%	37
	Zion Medpharma	200 mg.mL^−1^	<0.2%	37
	Herbarium	43 mg.mL^−1^	2.01 mg.mL^−1^	30
	L. Aura Farma	200 mg.mL^−1^	<0.2%	37
	Ease Labs	79.14 mg.mL^−1^	<0.2%	NI
	Extrato de *cannabis sativa* Cannten 200 mg.mL^−1^	200 mg.mL^−1^	<0.2%	37

NA, Not applicable; NI, Not informed.

Anvisa website accessed on April 27, 2023.

The terminology used in cannabinoid medicine also presents challenges. A comprehensive understanding of these terms is essential for clarity regarding the substances, the various types, and products available, and to prevent misuse. Table 2 outlines some important terms and definitions.^1^^,^^9^^,^^10^

**Table 2:** Definitions and terms used in cannabinoid medicine.^9^^,^^10^

***Cannabis sativa***: A plant from the Cannabaceae family (*Magnoliopsida urticales*) yields active components with varying concentrations and proportions, showcasing pharmacological effects. It is crucial to underline that cannabis is a heterogeneous plant, comprising various chemical constituents and types of phytocannabinoids.
**Marijuana**: Commonly referred to as “maconha” in Portuguese, denotes the cannabis plant without its isolated components.
**Cannabis**: This is the technical term for marijuana, referring to the plant itself without any specific medicinal application.
**Medicinal cannabis**: This term pertains to the therapeutic utilization of both the plant and its components for medical treatments.
**Endocannabinoids**: Endogenous ligands found in humans and other animals, possessing affinity and activity on cannabinoid receptors.
**Cannabinoids**: Plant components that activate receptors distributed throughout the body. Cannabinoids can exist in natural forms, referred to as phytocannabinoids, or they can be semi-synthetic or synthetic.
**Phytocannabinoid**: Cannabinoids naturally occurring in the plant.
**Cannabidiol (CBD)**: Major cannabinoid found in the cannabis plant, exhibiting pharmacological effects without producing psychoactive effects.
**Cannabinoid acids, cannabinol, cannabigerol, and cannabivarins**: Phytocannabinoids that demonstrate pharmacological properties but do not induce psychoactive effects.
**Tetrahydrocannabinol (delta-9-tetrahydrocannabinol – THC)**: Cannabinoid with psychoactive properties and primarily responsible for the majority of the effects associated with cannabis.
**Terpenes and flavonoids**: These are aromatic oils naturally occurring in the plant, responsible for imparting the characteristic smell and flavor unique to each plant, as well as contributing to its overall quality.
**Clone**: A selected plant chosen for replication, wherein a portion of the plant is cultivated to produce a new plant with identical genetic characteristics. This process is utilized for the selection and production of products intended for medicinal use.
**Full spectrum**: This refers to a product that contains all elements of the cannabis plant, providing what is known as the entourage effect, where multiple elements synergistically interact. The product may predominantly contain CBD or THC or be balanced, depending on the proportion of cannabinoids in its formulation.
**Broad spectrum**: A product that includes all elements of the cannabis plant except THC. It is recommended when THC is contraindicated.
**Isolated product**: A product containing only one component of the plant, with other cannabinoids removed.

Thus, it is vital to review the evidence on the efficacy and safety of cannabinoids in pain management due to the sizable number of products available for prescription and the vast existing literature. Therefore, a task force was established by the São Paulo State Society of Anesthesiology (SAESP) to examine the existing evidence related to cannabinoid-based product use for pain management.

## Consensus working method

The present narrative review, which involved anesthesiologists’ experts in pain management, used the Delphi method to achieve consensus. All pain specialists who are members of the SAESP Pain and Palliative Medicine Committee were invited to participate in the study panel. Additionally, renowned experts from several Brazilian states, totaling seventeen members, joined the panel. Initially, the group convened virtually to discuss the working method and determine the search mechanism to access the highest quality of evidence in the literature.

To establish consensus in the Delphi rounds, experts evaluated the themes by scoring them on a Likert scale from 1 to 5 (1 – Completely disagree; 2 – Disagree; 3 – Neither agree nor disagree; 4 – Agree; 5 – Totally agree). Scores of 1 and 2 were interpreted as disagreement, while scores of 4 and 5 were considered agreement. Panelists were allowed to freely enter their considerations, comments, and observations into the text. Identical weight was assigned to all individual opinions. To perform this review, an agreement coefficient higher than or equal to 60% was sought among the panelists to determine consensus.^11^

The members of the working group initially decided to include only review articles with meta-analysis available in the National Library of Medicine (PubMed) database. For this purpose, we utilized the search strategy outlined in Table 3.^2^ Studies addressing pain management were included, without restriction on the pathophysiology of pain, publication date, or language. However, only five meta-analyses were identified (Table 4). Subsequently, the working group opted to incorporate additional articles, provided that the subject matter addressed in the present review had not been covered in the content of the initially included publications.

**Table 3:** Strategy used for article search.

('guideline' [All Fields] AND 'systematic review' [All Fields]) AND 'chronic pain') [All Fields] AND 'cannabis' [All Fields] OR 'marijuana' [All Fields] OR 'hashish' [All Fields] OR 'cannabinoids' [All Fields] OR 'dronabinol' [All Fields] OR 'marinol' [All Fields] OR 'nabilone' [All Fields] OR 'cesamet' [All Fields] OR 'tetrahydrocannabinol' [All Fields] OR 'cannabidiol' [All Fields] OR ('nabiximols' [Supplementary Concept] OR 'nabiximols' [All Fields] OR 'sativex' [All Fields]) AND 'OR' [All Fields] AND ('nabiximols' [Supplementary Concept] OR 'nabiximols' [All Fields])

**Table 4:** Studies incorporated into the review following the consideration of the search strategy.

Aviram, Joshua, and G. Samuelly-Leichtag. “Efficacy of cannabis-based medicines for pain management: a systematic review and meta-analysis of randomized controlled trials”. Pain physician 20.6 (2017): E755.^5^
Meng, Howard, et al. “Selective cannabinoids for chronic neuropathic pain: a systematic review and meta-analysis”. Anesthesia & Analgesia 125.5 (2017): 1638-1652.^12^
Haeuser, Winfried, et al. “Efficacy, tolerability and safety of cannabis-based medicines for cancer pain: A systematic review with meta-analysis of randomized controlled trials”. Schmerz (Berlin, Germany) 33.5 (2019): 424-436.^13^
Dykukha, Igor, et al. “Nabiximols in chronic neuropathic pain: a meta-analysis of randomized placebo-controlled trials”. Pain Medicine 22.4 (2021): 861-874.^14^
Bilbao, Ainhoa, and Rainer Spanagel. “Medical cannabinoids: A pharmacology-based systematic review and meta-analysis for all relevant medical indications”. BMC medicine 20.1 (2022): 259.^15^

After assigning tasks among the authors of this review and distributing the aforementioned texts to be evaluated by the panelists, the first Delphi round was conducted electronically,^16^ involving all members of the working group. The initial consensus was reached with approximately 80% agreement, although some divergences persisted. Subsequently, a face-to-face meeting was convened with the participation of 15 authors to discuss the highlighted disagreements in the text.

One month after the initial meeting, the second Delphi round was conducted, also electronically, with the participation of all authors. During this round, approximately 90% agreement among authors was achieved. Additionally, a second face-to-face meeting was convened to finalize the manuscript. The recommendations provided in the present text are directed towards physicians who intend to prescribe medicinal cannabis to treat pain, as well as for physicians treating patients interested in receiving such prescriptions. This review focuses on pain scenarios for which data from the literature are available regarding the use of cannabinoids in pain management, as outlined below.

## Results

### Cannabinoids in cancer pain management

Experimental data from acute and chronic pain models have demonstrated synergistic analgesic effects between cannabinoids and opioids. Colocalization of cannabinoid and opioid receptors has been observed in the brain and spinal cord areas involved in pain modulation. Additionally, the release of endogenous opioid precursors has been noted following cannabinoid use. Therefore, it would be reasonable to expect a beneficial effect from the combined use of cannabinoids and opioids. However, clinical research in humans remains inconclusive regarding the benefit of combining cannabinoids and opioids for the management of cancer-related pain.^17^

A previous study observed only a modest analgesic effect in patients experiencing cancer-related pain who were administered daily doses of 10 or 20 mg of THC. The 10 mg dose was well tolerated, and despite the sedative effects, it exhibited analgesic potential. However, the 20 mg dose resulted in drowsiness, dizziness, ataxia, and blurred vision.^18^ Despite these promising results, subsequent randomized clinical trials failed to replicate the findings of the study.

A placebo-controlled study conducted by Johnson et al. (2010) reported the superiority of THC:CBD solution in reducing pain intensity; however, no reduction in opioid consumption was observed. Moreover, a higher incidence of nausea and vomiting was also observed with THC:CBD compared to the placebo group.^19^ These findings were not replicated in three other similar studies. Furthermore, Lynch et al. (2014) did not demonstrate the usefulness of nabiximols (synthetic cannabinoid) in managing pain related to chemotherapy-induced neuropathy.^20^

In a more recent review, the administration of nabiximols and THC via oral mucosa did not show significant differences from placebo in terms of reducing pain intensity, improving sleep, reducing opioid consumption, or incidence of adverse events. However, more patients reported improvement in general status with nabiximols and THC compared to placebo, although the Number Needed to Treat (NNT) was 16. Conversely, the rates of withdrawal from the study due to adverse events, represented by a Number Needed to Harm (NNH) of 20, indicated a higher occurrence with nabiximols and THC than with placebo. In summary, in this study, the NNT was very close to the NNH, rendering the clinical use of cannabinoids inadvisable in this scenario.^13^

In another review, the authors observed a reduction in pain intensity, improvement in sleep quality, and a reduction in opioid use among patients with cancer-related pain. The studies evaluated the effectiveness of THC and CBD-based treatments, with doses ranging from 2.7 to 43.2 mg/day of THC and 0 to 40 mg/day of CBD. Higher doses were associated with a higher incidence of side effects, including mental confusion (60%–70%), drowsiness (70%–100%) and euphoria (40%–50%). The most common side effects included fatigue, dry mouth, dizziness, and nausea, followed by drowsiness, hypotension, mental confusion, nausea, and vomiting.^2^^,^^21^

However, following a quantitative analysis of the literature, cannabinoids were not found to be superior to placebo in alleviating pain in cancer patients. Supporting these findings, the Multinational Association of Supportive Care in Cancer (MASCC) recently published a guideline based on a literature review. In their review, the authors stressed that no clinical study has revealed a reduction in opioid consumption by using cannabinoids for cancer-related pain management. Thus, MASCC does not recommend prescribing cannabinoids for cancer-related pain, further suggesting that the possible risk of harm and adverse events should be carefully considered for cancer patients.^2^^,^^22^

**Recommendations**: The authors of the present review do not recommend cannabinoids for the management of cancer-related pain.

### Cannabinoids in neuropathic pain management

A meta-analysis review that included patients presenting neuropathic pain, compared oral spray administration of CBD:THC to placebo. The authors of the meta-analysis observed pain intensity relief beyond that achieved with conventional analgesic strategies, suggesting potential usefulness for neuropathic pain management. Theoretically, CBD exhibits immunomodulatory and neuroprotective activities, which could contribute to neuropathic pain management. According to the authors’ analysis, the use of CBD and THC should be regarded as a third-line option.^14^

Wallace et al., in 2020, reported that both low or high doses of THC did not reduce pain intensity, whereas doses falling within a hypothetical and subjective therapeutic window led to pain intensity reduction in cases of pain related to diabetic peripheral neuropathy. In their study, aerosol doses of THC at proportions of 1%, 4%, or 7% were compared to placebo in terms of pain intensity and cognition after 4 hours. Additionally, blood samples were collected to quantify plasma THC concentration at various time points (0, 15, 30, 45, 60, 150, and 240 minutes after administration). Low and high plasma levels of THC did not result in pain reduction, while plasma concentrations between 16 and 31 ng.mL^−1^ induced analgesia. This effect could potentially be attributed to the expression of the Cannabinoid receptor type 1 (CB1) in the presence of nerve injury. Thus, the CB1 receptor may be considered a novel therapeutic target for analgesia in cases of diabetic peripheral neuropathy. Cannabinoid receptor type 2 (CB2), known to be abundantly found peripherally, represents another potential target for the management of pain related to neuronal injuries.^23^

A meta-analysis involving patients with neuropathic pain compared selective cannabinoids, such as dronabinol and nabilone, with conventional treatments (pharmacotherapy and/or physiotherapy) and placebo. This meta-analysis revealed a reduction in pain when cannabinoids were used; however, the decrease in pain intensity did not show a statistical difference. Despite this finding, the authors concluded that cannabinoids represent an important option for patients who are refractory to conventional neuropathic pain management or for those who are unable to adhere to conventional treatment due to side effects. Moreover, patients demonstrated improvements in sleep quality and overall quality of life without experiencing major adverse effects.^12^

The Canadian Pain Society categorizes selective cannabinoids as a third-line option for neuropathic pain management, whereas the International Association for the Study of Pain (IASP) underscores that there is insufficient evidence to fully support their use. However, IASP recommends against withdrawing their use for patients who show improvement, suggesting instead that supervision by a specialist is warranted.^24^^,^^25^

**Recommendations**: The authors of the present review recommend that cannabinoids should only be prescribed by specialists and considered as a third-line option for managing neuropathic pain.

### Cannabinoids in nociceptive pain management

#### Musculoskeletal pain

Musculoskeletal pain was previously a common reason for prescribing medicinal cannabis for adults.^26^ In a systematic review comprising 11 studies, seven noted a significant analgesic effect of cannabinoids, along with improvements in secondary pain-related symptoms, such as sleep patterns, muscle stiffness, and spasticity. The most common adverse effects were mild to moderate fatigue and dizziness, which were generally well tolerated.^27^

A recent scoping review underscored the scarcity of clinical trials studying the analgesic effect of cannabinoids on musculoskeletal pain. Conversely, it revealed that available publications reported improvements in pain scores and secondary symptoms, such as psychological well-being and a reduction in the consumption of analgesics, particularly opioids and adjuvants for pain management. The authors cautioned about the short follow-up in the included studies, as well as the observed methodological limitations.^26^

**Recommendations**: The authors of the present review recommend weighing the potential benefits and adverse effects before prescribing cannabinoids for the management of musculoskeletal pain, given their narrow therapeutic effective plasma concentration range.

#### Osteoarthritis

Osteoarthritis, the most prevalent musculoskeletal disorder, manifests primarily through pain. The expression of CB1 and CB2 receptors in the synovium suggests the involvement of the endocannabinoid system in this condition. Nonetheless, inhibition of the fatty acid amide hydrolase enzyme did not produce analgesic effects in patients with knee osteoarthritis.^27^ Thus, the prescription of cannabinoids for the disorder remains questionable.

In a 12-week randomized clinical trial involving 320 participants with a mean age of 69 years, topical CBD was applied to the skin adjacent to the knees of osteoarthritis patients. Participants were assigned to three groups: a placebo group and two groups using 4.2% CBD gel at doses of 250 mg/day and 500 mg/day. The primary analysis did not reveal any changes in pain scores. However, the secondary analysis showed a reduction of more than 30% in physical function assessed by the Western Ontario and McMaster Universities Arthritis Index. Male patients exhibited better responses than females, and dry mouth and headache were the most common adverse effects.^28^ Conversely, applying synthetic CBD (nabiximols) for 12 weeks in 136 individuals with hand osteoarthritis did not improve pain scores, sleep quality, or mood disorders.^28^ Clinical trials investigating oral or topical CBD, either alone or in combination with THC, for osteoarthritis management are currently underway, with most of them in phase 2.

**Recommendations**: The authors of the present review do not consider the existing evidence sufficient to support the routine prescription of cannabinoids for pain management in patients with osteoarthritis.

### Cannabinoids in nociplastic pain management

#### Fibromyalgia

Malfunctioning of the endocannabinoid system is believed to contribute to the pain experienced by patients with fibromyalgia. The endocannabinoid system plays a role in modulating inflammation, the endocrine system, cognition, memory, nausea, vomiting, and pain itself. Consequently, cannabinoids are theoretically considered a potential management option.^29^

However, several studies utilizing nabilone and dronabinol for fibromyalgia management reported a significant incidence of adverse effects, resulting in low tolerability to cannabinoids, poor treatment adherence, and unfavorable outcomes. Recent research with nabilone and varying proportions of THC and CBD also revealed unsatisfactory results.^30^

In a systematic review that included only two clinical trials involving 72 patients and with a short follow-up period, the tolerability of the cannabinoid used was low, with reports of dizziness, drowsiness, and vertigo. Furthermore, there was no improvement in fibromyalgia symptoms compared to placebo.^30^ When inhaled cannabis was administered to patients with fibromyalgia, no significant effects were observed in spontaneous pain or sensitivity to electric shock.^31^ According to the authors, the THC:CBD ratio might have influenced the outcomes, suggesting that THC may have a more suitable profile for pain management in patients with fibromyalgia.^30^^,^^31^

A clinical trial using THC and CBD sublingually at the mean dose of 4.4 and 0.08 mg, respectively, showed a significant reduction in fibromyalgia impact scores, and improved well-being, pain, and fatigue. Observational studies administering cannabinoids via different routes reported improvements in pain, quality of sleep, or quality of life. However, most patients included in these studies had previously used cannabinoids recreationally, and the mental effects linked to the substance may have influenced the results. The most commonly reported adverse effects were drowsiness, dry mouth, cough, and gastrointestinal symptoms, along with improvements in mood and libido as accompanying effects.^32^, ^33^, ^34^, ^35^

**Recommendations**: In fibromyalgia, the authors of the present review do not consider the existing evidence sufficient to recommend the routine prescription of cannabinoids. They also caution about the incidence of adverse effects associated with cannabinoid treatment in patients with fibromyalgia.

#### Visceral pain and chronic pelvic pain management

Experimental models of pancreatitis, esophagitis, hepatitis, and cystitis have shown that the stimulation of cannabinoid receptors exhibits tissue protective effects alongside analgesic and anti-hyperalgesic properties.^36^

The number of randomized and controlled clinical trials addressing irritable bowel syndrome surpasses those focused on inflammatory bowel disease. These studies suggest that while cannabinoids do not promote disease remission, they do alleviate pain intensity, anxiety, and depression, decrease opioid usage, thus mitigating their side effects, and ultimately enhance overall quality of life.^37^, ^38^, ^39^ However, routine clinical use of cannabinoids for these disorders should not be recommended.

Regarding male and female chronic pelvic pain, few observational studies support the cannabinoid prescription, rendering the results questionable.^40^, ^41^, ^42^ Most of the current literature comprises retrospective or cohort studies using different products, administration routes, and doses.^43^

**Recommendations**: The authors of this review do not recommend the routine administration of cannabinoids for visceral pain and chronic pelvic pain management.

#### Headaches and orofacial pain management

In a meta-analysis comprising 12 studies, which included case reports and series involving 1,980 participants, various doses of cannabinoids were administered using different administration routes aiming to evaluate their effects on abortive and preventive treatment of migraine. Individuals taking cannabinoids experienced a significant reduction in the number of episodes per month and in the occurrence of nausea and vomiting during episodes. However, this improvement did not persist beyond the sixth month of use. CBD:THC products showed a similar effect to amitriptyline in reducing the number of episodes per month. Combining both drugs (amitriptyline + cannabinoids) had an additive effect in reducing episodes, although moderate side effects were seen in 43.75% of patients, including tolerance and the subsequent need to increase doses.^44^ There are limited studies on migraine and other headaches to support the routine prescription of cannabinoids.

Similarly, there is a scarcity of clinical trials assessing cannabinoids for orofacial pain. In a systematic review, oral administration of cannabinoids as a preventive approach for orofacial procedures did not demonstrate superiority over ibuprofen or naproxen. However, lower analgesic consumption was observed in the cannabinoid group during the first eight hours, albeit without statistical significance. When pain was compared during the mobilization and the rest of the jaw, cannabinoid and placebo groups also showed no significant difference. Topical application of cannabinoids in the masseter area significantly reduced pain compared to placebo, accompanied by a reduction in electromyographic activity and pain at rest.^45^

Likewise, few clinical trials have investigated cannabinoids for nociplastic conditions and primary chronic pain. A systematic review encompassing acute and chronic facial pain included five studies with a total of 288 participants. These studies showed heterogeneity in terms of cannabinoid formulations, administration routes, target populations, and diagnosis, ranging from nociplastic pain and cancer-related pain to neuropathic pain.^46^

**Recommendations**: Although cannabinoids show promise in managing headaches and orofacial pain conditions, the current evidence is insufficient to justify their routine use.

#### Cannabinoids in acute pain management

There are more studies investigating cannabinoids for chronic pain than for acute pain management, leading to limited published evidence supporting their use. Gazendam et al. conducted a systematic review and meta-analysis in 2020 which included six randomized, placebo-controlled clinical trials with 678 participants.^6^ They found a statistically significant reduction in subjective pain scores in the cannabinoid groups compared to placebo groups for participants with acute postoperative pain, although the clinical significance was negligible. Intriguingly, pain reduction was observed only with the intramuscular route of administration, which is not widely available in many countries. However, the review had limitations, including variations in the overall quality and number of trials, as well as inconsistencies in reporting results. There was significant heterogeneity among the studies, including variations in cannabinoid type, dosage, duration, and administration route.^6^

In another meta-analysis published in 2020, evaluating cannabinoids for acute postoperative pain management across different types of surgeries, no significant difference was found in pain intensity or oral morphine consumption compared to control groups. Surprisingly, patients who received cannabinoids reported higher pain scores at 12 hours postoperatively, and they were also more likely to experience postoperative hypotension.^47^ Similarly, a 2017 systematic review and meta-analysis revealed that placebo groups achieved better outcomes compared to cannabinoid groups in terms of efficacy and adverse event incidence for postoperative pain.^5^

**Recommendations**: The authors of the present review do not recommend cannabinoids for acute pain management.

### Adverse effects, contraindications, and pharmacological interactions

The incorporation of cannabis-based products into clinical practice requires careful consideration by prescribing physicians, respecting individual clinical responses, adverse effects, and potential pharmacological interactions.^48^ THC is responsible for most of the therapeutical and adverse effects of cannabis, including its psychostimulant properties, whereas CBD lacks psychostimulant effect.^48^^,^^49^ Extreme caution is warranted when administering cannabis-based products to patients with specific genetic predispositions or psychiatric disorders such as psychosis, bipolar disorder, panic syndrome, anxiety, phobias, paranoia, abnormal liver or kidney function, amotivational syndrome of adolescence, and schizophrenia.^50^

Common adverse effects associated with THC include anxiety, panic syndrome, drowsiness, dry mouth, euphoria, hilarity, relaxation, and abnormal perception of distances. High doses may induce fear, agitation, psychotic manifestations, and impairments in attention and memory. Notably, even low doses can lead to adverse effects in individuals with increased sensitivity. Conversely, adverse effects associated with CBD are typically limited to changes in stool consistency, drowsiness, and hypotension.

Adverse effects are generally dose-dependent and can be mitigated by titrating doses accordingly.^51^ Most contraindications to cannabinoids are dose-dependent and primarily related to THC (Table 5). Although data on pharmacological interactions with medicinal cannabis are limited, it is metabolized by cytochrome CYP450 in the liver, which is also the metabolic pathway for several other drugs. CYP450-inducers like rifampicin reduce the maximum plasma concentration and area under the curve of THC and CBD, while inhibitors such as ketoconazole elevate the area under the curve relationship.^52^ Therefore, caution is advised to avoid potential interactions.

**Table 5:** Group of individuals and clinical scenarios requiring caution when prescribing medicinal cannabis.

Individuals below 25 years of age (relative contraindication): evidence indicates that high daily doses of THC can affect cognitive development, which continues until this age. It is associated with diminished motivation and poor academic performance.^50^
Personal history of substance abuse: presence of adult substance use disorder.^50^^,^^52^
Patients with psychiatric disorders: THC is contraindicated due to the risk of triggering crises in cases of moderate to severe disorder or poorly controlled disorder.^49^^,^^51^
Uncontrolled heart disorder: THC has been observed to increase heart rate.^53^ Additionally, patients who have used inhaled cannabis show a higher risk of perioperative acute myocardial infarction within the first 1 to 2 hours. When considering other routes of cannabinoid administration (non-inhalation), it is crucial to evaluate the risks and benefits before proceeding with elective surgery due to the temporal association of cannabis use and adverse cardiovascular effects.^54^ A study examined the risk of visiting cardiovascular emergency services and hospital admission among 18,653 adult patients authorized to use medicinal cannabis in Ontario, Canada, from 2014 to 2017. Medicinal cannabis was associated with an increased risk of emergency room visits or hospital admission related to cardiovascular events, including stroke and acute coronary syndrome.^55^
Patients with uncontrolled respiratory disorders: inhaled use is contraindicated for these patients. It is important to note that inhalation, or even vaporization, is prohibited in Brazil and should never be recommended by a physician. However, there are vaporizers approved for medicinal use in other countries.^53^
Pregnancy and lactation: there are a lack of safety data supporting the use of medicinal cannabis in these scenarios. Reports have detailed instances of newborns born to mothers who chronically used smoked cannabis, resulting in lower-than-average birth weights. In animal studies, THC has been observed to pass through the placenta, resulting in fetal plasma levels of approximately 10% of maternal levels after acute exposure, with significantly higher concentrations noted after repetitive exposure.^56^ Wide variations in cannabinoid concentrations in breast milk have been documented, persisting for an extended period and detectable up to six days after maternal consumption.^57^
Personal history of liver dysfunction: there may be an increase in liver enzymes with the use of CBD in patients who have a personal history of liver disorders. Therefore, it is crucial to identify patients with a history of liver disease and to conduct liver function tests before initiating CBD treatment, with subsequent testing every 3 months.^58^

In theory, THC may reduce serum concentrations of clozapine, haloperidol, duloxetine, olanzapine, cyclosporine, cyclobenzaprine, and theophylline, as they compete for the same metabolic pathway.^48^^,^^51^^,^^52^ Conversely, CBD could increase serum concentrations of haloperidol, antipsychotics, tricyclic antidepressants, calcium channel blockers, atorvastatin and simvastatin, beta-blockers, antihistamines, antiretrovirals, opioids, clobazam, macrolides, sildenafil, cyclosporine, tamoxifen, and warfarin.^49^^,^^59^^,^^52^ Most reported pharmacological interactions are associated with the concomitant use of CNS depressants, such as alcohol and benzodiazepines. Pimozide stands as an absolute contraindication for concurrent use with medicinal cannabis due to an increased risk of QT interval widening (Supplementary Material).^48^^,^^51^, ^52^, ^59^, ^60^

Other potentially harmful pharmacological interactions occur with checkpoint inhibitor immunotherapy for cancer treatment. Tumoral cells express cannabinoid receptors that can act as suppressors of tumor growth and metastases, or conversely promote neoplastic growth and present metastatic potential. Currently, immunotherapy employing inhibitors of various targets is an essential treatment for some histological tumor types. Since cannabinoids possess immunosuppressive properties, they can hinder the response of immunotherapeutic drugs. Studies have shown a decrease in time to tumor progression, reduced survival, or both, in cancer patients treated with cannabinoids and checkpoint inhibitors.^61^^,^^55^

It is important to note that liver function tests are recommended for all patients with a history of previous or active liver disorder. These tests should include measurements of aspartate Aminotransferase (AST), alanine Aminotransferase (ALT), bilirubin, gamma-glutamyl transferase, alkaline phosphatase, lactic dehydrogenase, total proteins, and prothrombin time. Clinical monitoring should be conducted grounded on baseline test results, with tests repeated every three months. If any test results show changes, the cannabinoid treatment should be halted, and the underlying causes should be investigated.^48^^,^^51^^,^^52^^,^^62^

Additionally, it is worth emphasizing that the CYP2D6 enzyme metabolizes many antidepressants. Therefore, CBD can increase serum concentrations of selective neurotransmitter reuptake inhibitor antidepressants, tricyclic antidepressants, antipsychotics, beta-blockers, and opioids, including codeine and oxycodone.^63^^,^^64^ Regular assessment of the patient and gradual dose adjustment are crucial. This approach allows for the monitoring of adverse effects and ensures the adherence to the pharmacovigilance plan. Treatment should be discontinued if there is no effectiveness, persistent adverse effects, or non-compliance with agreements previously established between the physician and the patient.^10^^,^^33^^,^^53^

In summary, monitoring liver function is clinically essential, especially in cases of pre-existing liver disorders. Moreover, the immunosuppressant effects of cannabinoids should be considered, particularly concerning cancer patients undergoing checkpoint inhibitor therapy, as they may negatively impact treatment outcomes.

## Conclusions

The risk-benefit assessment of cannabinoid use will evolve over time, alongside the determination of appropriate doses and its impact on pain relief, functional recovery, and quality of life.^65^ Cannabinoids can serve as allies in pain management when used judiciously. However, this task force does not recommend cannabinoids for the treatment of acute pain and cancer-related pain. In other clinical scenarios, currently recommended treatments should always be the first choice, especially in cases where published protocols exist, such as neuropathic pain. Only patients with inadequate clinical response or intolerance to recommended treatments should be considered as potential candidates for cannabinoids, which should be prescribed solely by specialists experienced in handling these substances.

Special attention should also be given to patient characteristics, particularly regarding the presence of pre-existing mental illnesses and the concurrent use of other drugs that may lead to potential drug interactions. Like other treatment modalities, cannabinoid therapy should be discontinued if it proves ineffective, if persistent adverse effects arise, or if the patient fails to comply with previously established agreements between physician and patient.

## Conflicts of Interest

The authors declare no have conflicts of interest.

## Appendix Supplementary materials

Image, application 1

Image, application 2

## Footnotes

Supplementary material associated with this article can be found in the online version at doi:10.1016/j.bjane.2024.844513.
