# Supplementary material for: Cannabinoid products for pain management: recommendations from the São Paulo State Society of Anesthesiology
Source: Braz J Anesthesiol. 2024 May 11;74(4):844513. doi: 10.1016/j.bjane.2024.844513 (PMC11167254; doi:10.1016/j.bjane.2024.844513)
Supplement: Supplementary file 1 [file mmc1.docx]

O uso de substâncias canabinoides no tratamento da dor: recomendações da Sociedade de Anestesiologia do Estado de São Paulo

*The use of cannabinoid compounds for pain management: recommendations by the Sao Paulo State Society of Anesthesiology*

*Running title: Cannabinoids for pain management.*

Resumo

Há crescente interesse no emprego de produtos canabinoides em diferentes situações clínicas, incluindo a dor. Este fato tem resultado em alguns países em negligência aos protocolos regulatórios. Lei promulgada no estado de São Paulo regulamenta a distribuição gratuita desses produtos para uso clínico. Nesse contexto, é importante que a literatura disponível acerca da eficácia e da segurança das substâncias canabinoides no tratamento da dor seja revista. Com esse objetivo, a Sociedade de Anestesiologia do Estado de São Paulo constituiu grupo de especialistas para realizar revisão narrativa com emprego de método Delphi, que considerou coeficiente mínimo para a determinação de consenso a concordância de 60% entre os autores. Incluíram-se inicialmente apenas artigos de revisão com metanálise, mas outras publicações poderiam ser consultadas, desde que o assunto abordado na presente revisão não estivesse contemplado no conteúdo disponibilizado pelos textos inicialmente selecionados. O estudo concluiu que os produtos canabinoides podem ser potenciais aliados no tratamento da dor, e que os mesmos devem ser empregados de forma judiciosa. Nas dores aguda e relacionada ao câncer, entretanto, esse grupo de autores não recomenda a sua utilização. Em outras situações clínicas o emprego dos tratamentos consolidados deve ser prioridade, em especial para situações em que existam protocolos pré-definidos, como na dor neuropática. Apenas pacientes com baixa resposta terapêutica, ou com intolerância aos tratamentos recomendados, podem ser eventuais candidatos ao emprego de canabinoides, que devem ser prescritos por especialistas com experiência no manuseio dessas substâncias. Atenção especial deve ser dada às características do paciente e à potencial ocorrência de interação medicamentosa.

Palavras-chave: Canabinoides; Dor; Resultado de tratamento; Revisão.

1. Introdução

Há interesse mundial no emprego de produtos canabinoides no tratamento de inúmeras doenças. A Organização das Nações Unidas (2020) reconheceu o potencial medicinal dos canabinoides e os excluiu do Anexo IV da Convenção Única sobre Entorpecentes, de 1961, o que permite seu emprego de maneira menos restritiva na clínica. ^1^ Na Europa, alguns países legalizaram o emprego medicamentoso de derivados vegetais da *Cannabis sativa* em várias situações, o que inclui o tratamento da dor, negligenciando os processos regulatórios habituais previstos para a comercialização de medicamentos. ^2^

O Estado de São Paulo sancionou, em 31 de janeiro de 2023, a lei 17.618 que institui política pública estadual para fornecimento gratuito de produtos canabinoides. No Brasil, esta é a primeira lei que prevê que os medicamentos e fitoterápicos contendo canabinoides sejam, de forma sistemática, fornecidos aos usuários do Sistema Público de Saúde. A lei contempla a disponibilização de medicamentos de origem vegetal à base de canabidiol (CBD), em associação com outras substâncias canabinoides, incluindo o tetrahidrocanabinol (THC). ^3^ Como consequência à promulgação da lei, grupo de trabalho foi constituindo pela Secretaria de Saúde do Estado contando com participação de representantes de distintas áreas da sociedade com interesse no tema. O principal objetivo do grupo de trabalho é a criação de protocolos para fornecimento dessas substâncias em diferentes situações clínicas, incluindo a dor.

No caso específico do tratamento da dor, apesar dos estudos mais recentes sinalizarem benefícios da cannabis medicinal, não existe evidência clínica robusta. Este fato se dá pela ocorrência de efeitos colaterais relacionados à sua prescrição, levando a um elevado índice de desistência dos participantes de estudos. Mas também pela variedade de produtos e concentrações administradas, relacionadas à grande heterogeneidade e inconsistência no momento de realização de metanálise, e eventualmente a sua inviabilização. ^4^

Acredita-se que a *Cannabis sativa* seja cultivada há mais 12.000 anos, embora o seu emprego como medicamento tenha ocorrido na China somente por volta de 2.700 AC. No mundo ocidental, o primeiro registro de sua utilização ocorreu em estudo pioneiro com seu emprego como analgésico, em 1839. Hoje, especialmente após os anos 1960, muitas pesquisas foram realizadas utilizando canabinoides como analgésico, como anti-inflamatório e na modulação da via nociceptiva sem, contudo, resultarem em evidências que autorizem sua prescrição rotineira. ^5^

Os motivos para a dificuldade em se obter evidências clínicas do emprego de canabinoides como analgésicos é a existência de barreiras práticas para a adequada realização de pesquisa. Entre elas, podem-se citar: ^4,6^

1. existência de medidas regulatórias excessivamente restritivas;

2. os produtos à base de cannabis disponíveis no mercado não são contemplados por fundos de pesquisa federais (mesmo em países desenvolvidos), o que dificulta o seu acesso, para fins de pesquisa;

3. as pesquisas publicadas têm a predominância de financiamento privado, o que gera vieses de resultados, fazendo com que seja urgente a criação de políticas públicas de incentivo à pesquisa com essas substâncias;

4. há grande variedade de fármacos e produtos disponíveis para comercialização.

São conhecidadas aproximadamente 530 substâncias químicas derivadas da *Cannabis sativa.* Destas, 100 compostos canabinoides foram isolados, o que representa enorme desafio para a obtenção de evidências científicas com o seu emprego clínico. ^7^ Durante a confecção desta revisão narrativa, 16 produtos contendo canabidiol isolado e 11 produtos contendo extrato da planta *Cannabis sativa* em diferentes proporções estavam aprovados pela Agência Nacional de Vigilância Sanitária (Anvisa) para venda em farmácias no Brasil (Tabela 1). ^8^ Vale ressaltar que as inúmeras vias de administração também adicionam maiores desafios à obtenção de evidências. Há estudos que se utilizam da via inalatória, potencialmente iatrogênica, para a administração dos compostos, o que não é recomendada pelo *Federal Drug Administration*, Estados Unidos da América, assim como pela Anvisa.

A terminologia empregada na medicina canabinoide também representa um desafio próprio. O adequado conhecimento dos termos é importante para o claro entendimento das substâncias, bem como dos tipos e produtos disponíveis e, também, para evitar o uso inapropriado. Alguns termos e definições importantes são citados no quadro 1. ^9,10^

Nesse contexto, de maior disponibilização de produtos canabinoides disponíveis para prescrição e a existência de muitos estudos disponíveis para consulta, é importante que se revise as evidências da eficácia e de segurança dos medicamentos canabinoides no tratamento da dor. Com esse objetivo foi constituído grupo de trabalho no âmbito da Sociedade de Anestesiologia do Estado de São Paulo (SAESP) para discutir as evidências disponíveis para o emprego de substâncias à base de canabinoides no tratamento da dor. Inicialmente este grupo se reuniu de forma virtual para debater o método de trabalho e o mecanismo de busca que seria empregado para acessar a melhor qualidade de evidência disponível na literatura.

2. Método de trabalho do consenso

Trata-se de uma revisão narrativa que contou com a participação de especialistas na área de tratamento da dor, todos anestesiologistas, com emprego de método Delphi para obtenção de consenso. Todos os especialistas em dor que fazem parte do Comitê de Dor e Medicina Paliativa da SAESP foram convidados para integrar os painelistas do estudo. A estes somaram-se outros renomados especialistas dos estados da Bahia, Maranhão, Minas Gerais, Rio de Janeiro e São Paulo, perfazendo o total de dezessete membros.

Para o estabelecimento de consenso, nas rodadas Delphi, os especialistas avaliaram os temas, pontuando-as em uma escala Likert de 1 a 5, sendo 1) discordo totalmente; 2) discordo; 3) não concordo nem discordo; 4) concordo; 5) concordo totalmente. São entendidos como discordância, 1 e 2; como concordância, 4 e 5. Aos painelistas foi permitido inserir no texto suas considerações, comentários e observações livremente. Foi dado igual peso para todas as opiniões individuais. Para a realização deste trabalho buscou-se um coeficiente de concordância maior ou igual 60% para a determinação de consenso. ^11^

Os membros desse consenso decidiram pela inclusão inicial apenas de artigos de revisão com metanálise que estivessem disponibilizados no banco de dados da *National Library of Medicine* (PubMed) com o emprego da estratégia de busca contida no quadro 2. ^2^ Foram incluídos estudos que abordam o tratamento da dor, sem restrição de sua fisiopatologia, data de publicação ou idioma. Com esta busca, encontraram-se cinco publicações (Quadro 3). O grupo de trabalho decidiu também incluir outras publicações, desde que o assunto abordado pela presente revisão não estivesse contemplado no conteúdo disponibilizado pelas metanálises inicialmente incluídas.

Após a distribuição de tarefas entre os autores desta revisão, e a entrega dos referidos textos a serem avaliados pelos painelistas, foi realizada a primeira rodada Delphi por meio eletrônico. Nesta rodada houve participação de todos os integrantes do grupo de trabalho. ^12^ Obtido consenso inicial com concordância de aproximadamente 80%, embora ainda com algumas divergências, foi realizada reunião presencial com a participação de 15 dos autores para que as divergências apontadas na redação fossem discutidas.

Um mês após esta primeira reunião realizou-se a segunda rodada Delphi, de forma eletrônica, com a participação de todos os autores. Nesta rodada obteve-se coeficiente de concordância entre os autores de aproximadamente 90%. Da mesma forma, uma segunda reunião presencial ocorreu com o objetivo de chegar à redação final do texto, que é aqui apresentado.

As recomendações contidas neste texto destinam-se aos médicos que intencionam prescrever cannabis medicinal para o tratamento de dores, assim como para aqueles que se deparam com o desejo dos doentes de receberem prescrição destas substâncias para as mesmas situações clínicas. Este trabalho aborda as situações de dores em que há dados disponíveis na literatura sobre o emprego de substâncias canabinoides nos seus tratamentos, como apresentado a seguir.

3. Dor oncológica

Dados experimentais em modelos de dor aguda e crônica têm demonstrado ocorrência de efeitos analgésicos sinérgicos entre os canabinoides e os opioides. Existe colocalização dos receptores canabinoides e opioides em áreas do cérebro e da medula espinhal envolvidas nas vias modulatórias da dor. Observa-se, também, liberação de precursores de opioides endógenos pelo emprego de canabinoides. O efeito benéfico da associação dessas classes, portanto, seria esperado. Pesquisas clínicas em humanos, no entanto, ainda são pouco conclusivas em relação ao benefício da associação de canabinoides e de opioides no tratamento da dor relacionada ao câncer. ^13^

Em publicação de 1975, observou-se pequeno efeito analgésico em pacientes com dor relacionada ao câncer e que haviam recebido doses diárias de THC de 10 ou 20 mg. A dose de 10 mg foi bem tolerada e, apesar de seu efeito sedativo, mostrou potencial analgésico. Já a dose de 20 mg, induziu sonolência, tontura, ataxia e visão turva. ^14^ Apesar destes resultados favoráveis, outros trabalhos clínicos randomizados não conseguiram reproduzir este os achados deste estudo.

Em outro trabalho, conduzido por Johnson et al. (2010), observou-se superioridade na redução da intensidade da dor com emprego de nabiximol (canabinoide sintético) em relação ao THC, em pacientes recebendo solução de THC:CBD, quando comparados com placebo, mas não houve redução do consumo diário de opioide. Houve maior ocorrência de náuseas e vômitos com uso de THC:CBD em comparação com o grupo placebo. ^15^ Tais resultados não puderam ser reproduzidos por outros três trabalhos semelhantes, assim como Lynch et al. (2014) não demonstraram utilidade de nabiximol no alívio da dor relacionada à neuropatia induzida por quimioterapia. ^16^

Em revisão mais recente, o nabiximol e o THC administrados via mucosa oral não diferiram do placebo na redução da intensidade de dor, na melhora do sono, no consumo de opioides e na frequência de eventos adversos. Entretanto, o número de pacientes que relatou melhora do estado geral foi maior com nabiximol e com o THC do que com o placebo, mas o número necessário para um paciente se beneficiar (*number needed to treat -* NNT) foi de 16. As taxas de abandono no estudo em consequência da ocorrência de eventos adversos, ou seja, o número necessário para ocorrer um dano adicional (*number needed to harm* - NNH) foi de 20, maior com nabiximol e THC do que com placebo. Em resumo, nesta pesquisa o NNT é muito próximo ao NNH, o que torna o uso clínico de canabinoides desaconselhável neste cenário. ^17^

Em outra revisão, os autores observaram redução da intensidade de dor, melhora da qualidade do sono e redução do uso de opioides em pacientes com dor associada ao câncer. Os autores encontraram estudos avaliando a eficácia de terapias à base de THC e CBD, com doses variando de 2,7 a 43,2 mg/dia de THC e de 0 a 40 mg/dia de CBD. Os efeitos colaterais foram mais frequentes com doses maiores. Pode ocorrer confusão mental (60-70%), sonolência (70-100%) e euforia (40-50%). Efeitos colaterais como fadiga, boca seca, tontura e náusea foram os mais comuns. Secundariamente, ocorreram sonolência, hipotensão, confusão mental, náuseas e vômitos. ^2,18^

Todavia, em análise quantitativa da literatura, foi observado que os canabinoides não se mostraram superiores ao placebo no alívio da dor de pacientes com câncer. Reforçando esses dados, recentemente a *Multinational Association of Supportive Care in Cancer* (MASCC) publicou uma diretriz com base em revisão da literatura. Nesta revisão os autores apontam que nenhum trabalho clínico demonstrou redução do consumo de opioides com o uso de canabinoides em tratamento de dor relacionada ao câncer. Por este motivo, a MASCC não recomenda a prescrição de canabinoides para tratamento da dor no câncer, sugerindo, ainda, que o risco potencial de danos e eventos adversos sejam cuidadosamente considerados para todos os pacientes com câncer. ^2,19^

Desta forma, os autores da presente revisão não recomendam o emprego de substâncias canabinoides no tratamento da dor relacionada ao câncer.

4. Dor neuropática

Em revisão com metanálise, com a inclusão de pacientes com dor neuropática (DN), comparou-se a administração de CBD:THC em spray oral com placebo, concluindo-se que há alívio da dor adicional ao alcançado com estratégias analgésicas convencionais, podendo auxiliar no tratamento de DN. Teoricamente o CBD possui atividade imunomoduladora e neuroprotetora, podendo contribuir para o tratamento da DN. Segundo análise desses autores, o alívio da dor proporcionado pelo CBD e THC é adicional ao alcançado com estratégias analgésicas convencionais para DN, e considerada conduta de terceira linha. ^20^

Em publicação de Wallace et al. em 2020, demonstrou-se que doses baixas ou elevadas de THC não produziram efeito de redução na intensidade da dor, enquanto as doses dentro de uma suposta e subjetiva janela terapêutica resultaram em redução da intensidade da DN em pacientes com neuropatia periférica diabética dolorosa. Nesse estudo foram comparadas as doses de THC na proporção 1%, 4% ou 7% versus placebo, em aerossol, e avaliada a intensidade da dor e realizados testes cognitivos em 4 horas. Foram também colhidas amostras de sangue para quantificar a concentração plasmática de THC (0, 15, 30, 45, 60, 150 e 240 minutos após a administração). Níveis plasmáticos baixos e altos de THC não produziram redução na dor, enquanto concentrações plasmáticas compreendidas entre 16 e 31 ng.mL^-1^ resultaram em redução da intensidade de dor. Este fato pode ser, provavelmente, consequente à expressão do receptor canabinoide tipo 1 (CB1) que ocorre na presença de lesão nervosa. Assim, o receptor CB1 pode passar a ser considerado novo alvo para analgesia em casos de neuropatia periférica diabética. Sabe-se também que o receptor canabinoide tipo 2 (CB2) é encontrado em abundância na periferia, representando outro potencial alvo para o tratamento das dores associadas às lesões neuronais. ^21^

Metanálise realizada incluindo pacientes com DN que comparou canabinoides seletivos, como o dronabinol e a nabilona, tratamentos convencionais (farmacoterapia e/ou fisioterapia) e o emprego de placebo, demonstrou redução da intensidade da dor com o emprego de canabinoides. Mas essa diminuição da intensidade não apresenta diferença estatística. Apesar disso, os autores concluem que o emprego de canabinoides é uma possibilidade importante em pacientes refratários aos tratamentos preconizados da DN, ou para aqueles que não conseguem aderir aos tratamentos convencionais pela ocorrência de efeitos colaterais. Os pacientes também apresentaram incremento na qualidade do sono e na sua qualidade de vida, sem efeitos adversos significativos. ^22^

A *Canadian Pain Society* considera que os canabinoides seletivos são opção de terceira linha para a DN, enquanto a *International Association for the Study of Pain* destaca o fato de não haver evidências científicas suficientes para apoiar o uso dos canabinoides, porém orienta a não suspensão do seu uso em pacientes que estejam se beneficiando, e aconselha a supervisão de um especialista. ^23,24^

Baseados nesses trabalhos publicados, os autores deste artigo de posicionamento da SAESP recomendam que os produtos canabinoides sejam prescritos apenas por especialistas e como terceira linha de tratamento para pacientes portadores de DN.

5. Dores nociceptivas

5.1 Dor musculoesquelética

A dor musculoesquelética já foi a causa mais frequente para a prescrição da cannabis medicinal em adultos. ^25^ Em revisão sistemática que contou com a inclusão de 11 pesquisas observou-se, em sete delas, efeito analgésico significativo quando da utilização de canabinoides, além de melhora de sintomas secundários como sono, rigidez muscular e espasticidade. Os efeitos adversos observados com maior frequência foram fadiga e tontura, em gravidade leve a moderada, e geralmente bem tolerados. ^26^

Outra revisão de escopo recente destacou a falta de ensaios clínicos abordando a dor musculoesquelética de forma isolada, mas demonstrou que as publicações disponíveis evidenciam melhora nos escores de dor e de sintomas secundários, como bem-estar psíquico e redução no consumo de analgésicos, especialmente opioides, e de adjuvantes para tratamento da dor. Os autores, porém, alertam para o curto período de seguimento nos estudos incluídos, além das limitações metodológicas observadas. ^25^

Pelos dados apresentados, os autores deste artigo de posicionamento quanto ao emprego de substâncias canabinoides no tratamento da dor musculoesquelética recomendam ponderar entre os possíveis benefícios da prescrição dessas substâncias e os seus efeitos adversos, uma vez que esses limites são muito estreitos.

5.2 Osteoartrite

A osteartite é a doença musculoesquelética mais comum, sendo dor o seu principal sintoma. A expressão de receptores CB1 e CB2 na sinóvia, teoricamente, demonstra o envolvimento do sistema endocanabinoide nesta enfermidade. Observou-se, contudo, que a inibição da enzima amida hidrolase de ácidos graxos não produz efeitos analgésicos em portadores de osteoartrite em joelhos. ^26^ A prescrição de produtos canabinoides para a enfermidade segue, assim, questionável.

Em ensaio clínico randomizado com duração de 12 semanas, e com a inclusão de 320 indivíduos de idade média de 69 anos, aplicou-se CBD tópico em joelhos de pacientes com osteoartrite em três grupos: um grupo placebo e dois grupos com utilização de 250 mg/dia ou 500 mg/dia de CBD em gel 4,2%. Nesse trabalho, não se demonstrou alteração dos escores de dor na análise primária. Na análise secundária de redução do escore de dor intensa, observou-se melhora de mais de 30% da intensidade da dor, além de melhora de função física avaliada pelo índice da *Western Ontario and McMaster Universities Arthritis* (mais 20% de melhora). Pacientes do sexo masculino responderam melhor que as mulheres e a queixa de boca seca e de cefaleia foram os efeitos adversos mais relatados. O emprego de CBD sintético (nabiximol) em osteoatrite de mão, durante 12 semanas e em 136 indivíduos, entretanto não demonstrou melhora em escores de dor, na qualidade do sono e nos distúrbios de humor. ^27^

Ensaios clínicos com CBD oral ou tópico, associado ou não ao THC, em osteoartrite, estão em desenvolvimento, grande parte em fase 2. Desta forma, os autores do presente posicionamento não consideram as evidências disponíveis suficientes para a prescrição rotineira de produtos canabinoides para o tratamento da dor em pacientes com osteoartrite.

6. Dores nociplásticas

6.1 Fibromialgia

Na fibromialgia, supõe-se a ocorrência de falhas no sistema endocanabinoide como uma das possíveis explicações para a presença de dor. Sabe-se que o sistema endocanabinoide está envolvido na modulação da inflamação, do sistema endócrino, da cognição, da memória, da náusea, do vômito e da própria dor. Dessa forma, o emprego de canabinoides pode ser uma possibilidade teórica de opção terapêutica. ^28^

Contudo, vários trabalhos utilizaram nabilona e dronabinol no tratamento da fibromialgia e observaram significativa incidência de efeitos adversos e, consequentemente, baixa tolerabilidade aos canabinoides, baixa aderência ao tratamento e resultados desfavoráveis. Pesquisa posterior com nabilona e proporções variadas de THC e CBD também mostraram resultados não satisfatórios. ^29^

Em revisão sistemática que incluiu apenas dois ensaios clínicos, com 72 pacientes no total, e com seguimento por curto período, a tolerabilidade ao canabinoide empregado foi baixa e houve relato de tontura, sonolência e vertigem. Não houve melhora dos sintomas da fibromialgia em relação ao placebo. ^29^ Em emprego de cannabis inalado, nenhum tratamento reduziu a dor espontânea ou a sensibilidade ao choque elétrico mais que o placebo. ^30^ Segundo os autores, a relação THC:CBD pode ter influenciado no resultado. O CBD aumenta a concentração do THC, contudo antagoniza os efeitos analgésicos deste. Isso favorece a ideia de que o THC apresenta perfil mais favorável para o tratamento da dor no paciente com fibromialgia. ^29,30^

Ensaio clínico com emprego de THC e de CBD por via sublingual, no qual a dose média empregada foi de 4,4 e 0,08 mg, respectivamente, houve redução significativa dos escores do impacto da fibromialgia, melhora do bem-estar, da dor e da fadiga. Pesquisas observacionais, com canabinoides por diversas vias, relataram melhora da dor, da qualidade do sono ou da qualidade de vida, no entanto, a maioria dos pacientes já utilizaram esses agentes de maneira recreativa e os efeitos psíquicos vinculados à substância pode ter influenciado os resultados. Os efeitos adversos mais relatados, na maioria dos trabalhos, foram sonolência, boca seca, tosse e sintomas gastrointestinais, além da melhora do humor e da libido como efeitos concomitantes. ^31–34^

Também em fibromialgia, os autores do presente estudo não consideram as evidências disponíveis suficientes para a prescrição rotineira de produtos canabinoides, além de alertarem para a ocorrência de efeitos adversos relacionados a esta terapia nesses pacientes.

6.2 Dor visceral e dor pélvica crônica

Em modelos experimentais de pancreatite, esofagite, hepatite e cistite, foi demonstrado que a estimulação de receptores canabinoides apresenta efeitos protetores teciduais e propriedades analgésicas e anti-hiperalgésicas. ^35^

O número de ensaios clínicos randomizados e controlados em síndrome do cólon irritável é mais expressivo comparativamente do que em doença inflamatória intestinal. Esses trabalhos sugerem que, apesar dos canabinoides não serem responsáveis pela remissão da atividade de doença, eles reduzem a intensidade da dor, a ansiedade e a depressão, diminuindo o consumo de opioides, desta forma, mitigando efeitos colaterais e melhorando globalmente a qualidade de vida. ^36–38^ Contudo, não é possível recomendar, para uso clínico rotineiro, nessas situações.

Em dor pélvica crônica são poucos os estudos observacionais, tanto no homem como na mulher, com resultados questionáveis em favor dos produtos canabinoides. ^39–41^ A maioria da literatura atual é composta de pesquisas retrospectivas ou de coorte com diferentes produtos, vias de administração e posologias. ^42^

Por estes motivos, os autores deste atual trabalho não recomendam a utilização rotineira de canabinoides nessas situações clínicas.

6.3 Cefaleias e dor orofacial

Em metanálise com inclusão de 12 estudos (envolvendo séries de casos e relatos), com 1.980 participantes, foram utilizadas diversas doses de compostos canabinoides, podendo ou não incluir o THC, por diferentes vias de administração, com o objetivo de avaliar seus efeitos no tratamento abortivo e preventivo da migrânea. Houve redução significativa no número de crises por mês, de náuseas e de vômitos durante as crises. Contudo, essa melhora não persistiu após o 6º mês de uso. Quando comparados à amitriptilina, os produtos CBD:THC demonstraram efeito similar na redução do número de crises por mês. O uso associado desses fármacos (amitriptilina + canabinoides) demonstrou efeito aditivo na redução das crises. Efeitos colaterais moderados se apresentaram em 43,75% dos pacientes, ocorrendo inclusive tolerância e, consequentemente, necessidade de elevadas doses. ^43^ Poucas são as pesquisas em migrânea e demais cefaleias, para se justificar prescrição rotineira.

Poucos também são os ensaios clínicos com canabinoides em dor orofacial. Em revisão sistemática, o uso de canabinoide por via oral, preventivamente à realização de procedimentos orofaciais, demonstrou não haver superioridade ao uso de ibuprofeno e naproxeno, porém resultando em menor consumo de analgésicos nas primeiras oito horas, para o grupo canabinoide, mas sem significância estatística. Comparando a dor em movimento e repouso (mobilização da mandíbula), também não houve diferença entre o grupo canabinoide e placebo. O uso na apresentação tópica, em região de masseter, demonstrou redução significativa da dor, comparado ao placebo, com redução da atividade eletromiográfica e dor ao repouso. ^44^

Também, são poucos os ensaios clínicos que abordam este tipo de entidade nociplástica e dor crônica primária. Em revisão sistemática na qual cinco trabalhos foram selecionados, perfazendo o total de 288 participantes, foram avaliadas dor facial aguda e crônica. Os estudos incluídos nessa revisão possuiam heterogeneidade nas formulações de canabinoides utilizados, vias de administração, população abordada, além de diagnósticos diversos, desde dor nociplástica, à dor oncológica e à neuropática. Apesar do emprego de canabinoides aparentar efeitos positivos nesses quadros dolorosos, a evidência ainda é baixa e insuficiente para justificar o emprego rotineiro. ^45^

7. Dor aguda

Há mais publicações com emprego dos canabinoides no tratamento da dor crônica do que para o tratamento da dor aguda, o que confere ainda menos evidência na literatura ao emprego dessas substâncias. Gazendam et al., em 2020, publicaram uma revisão sistemática e metanálise, em que foram incluídos seis ensaios clínicos randomizados, controlados por placebo, com a inclusão de 678 pacientes. Nesta revisão, houve redução clinicamente não significativa, mas estatisticamente significativa, nos escores subjetivos de dor nos grupos canabinoides quando comparados ao placebo, em pacientes com dor aguda pós-operatória. Observou-se, também, diferença significativa no tamanho do efeito entre as vias de administração oral e não oral, com redução dos escores de dor somente pela via intramuscular, apresentação ainda não disponível no Brasil. No entanto, os autores colocam como limitações da pesquisa a qualidade e quantidade geral dos ensaios disponíveis, bem como pela inconsistência no relato de resultados. Houve heterogeneidade significativa nos estudos incluídos e variação no tipo, dosagem, tempo, duração e via do canabinoide usado. ^6^

Entretanto, em outra metanálise realizada, também em 2020, avaliou-se o emprego de canabinoides para o tratamento da dor aguda pós-operatória em diferentes tipos de cirurgias com base em oito ensaios controlados e randomizados e em quatro estudos observacionais. Nesta publicação, não foi encontrada diferença na intensidade da dor em repouso em 1, 6 ou 24 horas após o procedimento cirúrgico, ou no consumo acumulado de morfina por via oral em 2 ou 24 horas no período pós-operatório, quando comparado ao grupo controle. De forma surpreendente, os pacientes que receberam canabinoides tiveram maiores escores de dor, com diferença significativa em três manuscritos que avaliaram a dor 12 horas após a operação. Ainda, os pacientes que usaram canabinoides tiveram 3,24 vezes mais chances de desenvolver hipotensão no período pós-operatório. ^46^

Em outra revisão sistemática e metanálise, publicada em 2017, os autores apresentaram informações quanto à eficácia e à ocorrência de eventos adversos ao examinarem os efeitos na dor pós-operatória. Nesta, os resultados de três estudos controlados e randomizados mostraram que o grupo placebo apresentou resultados melhores em relação aos que usaram canabinoides. ^5^

Com base nos dados apresentados, os autores deste atual trabalho não recomendam a utilização rotineira de canabinoides para o tratamento de dores agudas.

8. Efeitos adversos, contraindicações e interações farmacológicas

A integração na prática clínica de produtos à base de cannabis deve ser realizada com cautela pelos prescritores médicos, de acordo com a individualidade de respostas clínicas, de efeitos adversos e possíveis interações farmacológicas. ^47^ O THC é responsável pela maioria dos efeitos farmacológicos e adversos da cannabis, incluindo seu efeito psicoestimulante. O CBD, em contrapartida, não apresenta efeito psicoestimulante. ^47,48^

Também deve-se exercer extrema cautela na administração desses produtos em pacientes com predisposição genética ou na presença de doença psiquiátrica, nas seguintes situações**:** psicose, transtorno bipolar, síndrome do pânico, ansiedade, fobias, paranoia, alterações de funções hepática ou renal, síndrome amotivacional da adolescência e em esquizofrenia. ^49^

Entre os efeitos adversos mais relatados, são citados: ^50^

- THC: ansiedade, síndrome do pânico, sonolência, boca seca, euforia, hilaridade, relaxamento, alteração de percepção de distâncias, predispondo à ocorrência de acidentes. Em doses altas, podem também ocorrer medo, agitação, manifestações psicóticas, problemas na atenção e memória. É importante ressaltar que pessoas com maior sensibilidade podem apresentar esses efeitos adversos mesmo com doses baixas;
- CBD: alteração da consistência das fezes, sonolência e hipotensão.

Esses efeitos adversos são geralmente dose-dependentes e, portanto, pode-se minimizá-los ao titular as doses. ^51^

As contraindicações ao emprego de substâncias canabinoides são dose-dependentes e quase todas relacionadas ao THC (Quadro 4). Apesar de existirem poucos dados sobre interação farmacológica com cannabis medicinal, sabemos que seu metabolismo se dá no fígado através do citocromo CYP450, a mesma via metabólica utilizada por muitos outros fármacos.

Não existem pesquisas específicas com o objetivo de estudar as interações farmacológicas com a cannabis, mas sim observações em ensaios com outros objetivos primários e secundários. As interações podem aumentar, ou mesmo diminuir, a concentração plasmática dos medicamentos em uso concomitante, o que é muito importante para o tratamento clínico dos pacientes em uso de outros fármacos. Os indutores do CYP450, como a rifampicina, diminuem a concentração máxima e a área sob a curva do THC e do CBD, enquanto os inibidores do CYP450, como o cetoconazol, aumentam esta relação. ^52^

Teoricamente, por competir pela mesma via de metabolização, o THC pode diminuir as concentrações séricas de clozapina, haloperidol, duloxetina, olanzapina, ciclosporina, ciclobenzaprina e teofilina. ^47,51,52^ Já o CBD pode aumentar as concentrações séricas de haloperidol, antipsicóticos, antidepressivos tricíclicos, bloqueadores de canal de cálcio, atorvastatina e sinvastatina, betabloqueadores, anti-histamínicos, antirretrovirais, opioides, clobazam, macrolídeos, sildenafila, ciclosporina, tamoxifeno e varfarina. ^48,50,52^

A maioria das interações farmacológicas estão associadas ao uso concomitante de depressores do SNC, como o álcool e os benzodiazepínicos. De todos os fármacos, somente a pimozida apresenta contraindicação absoluta ao uso concomitante com cannabis medicinal, pelo seu risco aumentado do alargamento do intervalo QT no eletrocardiograma (Material Suplementar). ^47,50–53^

Outras interações farmacológicas potencialmente danosas são as que ocorrem com a imunoterapia de inibidores de *checkpoint* no tratamento do câncer. Sabe-se que as células tumorais expressam receptores canabinoides que podem atuar como supressores do crescimento tumoral e da ocorrência de metástases, assim como podem promover o crescimento neoplásico e ter potencial metastático. Atualmente, imunoterapia com inibidores de diferentes alvos já é realidade, assim como, tratamento essencial para alguns tipos histológicos de neoplasia. Os canabinoides, como são imunossupressores, podem impedir as respostas dos imunoterápicos inibidores de *checkpoint* no tratamento do câncer. Estudos demonstraram uma diminuição no tempo de progressão do tumor, uma sobrevida reduzida, ou ambos, quando os pacientes oncológicos são tratados com substâncias canabinoides e um inibidor de *checkpoint*. ^54,55^

Com base nesses conhecimentos, recomenda-se a realização de exames de função hepática para todos os pacientes que referirem hepatopatia prévia ou em atividade, incluindo as dosagens de TGO, TGP, bilirrubinas, gama glutamiltransferase, fosfatase alcalina, desidrogenase lática, proteínas totais e tempo de protrombina. O acompanhamento clínico deve ser baseado nos resultados iniciais obtidos e nas repetições dos exames a cada três meses. Caso algum dos parâmetros esteja apresentando modificações, deve-se interromper o uso da substância canabinoide e investigar as causas. ^47,51,52,56^

Também deve-se atentar ao fato de que a enzima CYP2D6 metaboliza muitos antidepressivos. Portanto o CBD pode aumentar as concentrações séricas de antidepressivos inibidores seletivos de recaptação de neurotransmissores, de antidepressivos tricíclicos, de antipsicóticos, de betabloqueadores e de opioides, incluindo a codeina e a oxicodona. ^57,58^

A avaliação do risco-benefício se constrói ao longo do tempo, bem como o ajuste das doses e o impacto no alívio da dor, a recuperação funcional e a qualidade de vida. ^59^ É importante avaliar periodicamente o paciente e ajustar a dosagem gradualmente. Isso permite a monitoração de efeitos adversos e assegura o programa de farmacovigilância. O tratamento deverá ser descontinuado caso haja ausência de efetividade, efeitos adversos persistentes e não cumprimento dos acordos previamente firmados entre médico e paciente. ^10,32,60^

9. Conclusões

Os produtos canabinoides podem ser aliados no tratamento da dor, desde que empregados de forma judiciosa. No tratamento da dor aguda e da dor relacionada ao câncer, entretanto, esse grupo de autores não recomenda a sua utilização. Em outras situações clínicas, o emprego dos tratamentos já preconizados deve sempre ser prioridade, em especial para situações clínicas em que existam protocolos publicados, como na dor neuropática. Apenas pacientes com baixa resposta clínica, ou com intolerância aos tratamentos recomendados, devem ser eventuais candidatos ao emprego de canabinoides – somente prescritos por especialistas que tenham experiência no manejo dessas substâncias.

Deve-se, também, dar especial atenção às características do paciente, especialmente no que tange à pré-existência de doenças mentais e ao emprego concomitante de outros fármacos que podem ter potencial interação medicamentosa. Tal qual ocorre com outras condutas terapêuticas, o tratamento com canabinoides deve ser descontinuado na ausência de sua efetividade, na ocorrência de efeitos adversos persistentes e no caso de não cumprimento dos acordos previamente firmados entre médico e paciente.

**Conflitos de interesse e fontes de financiamento e agradecimento**

Os autores declaram não haver conflitos de interesse. Este estudo contou com o apoio logístico da Sociedade de Anestesiologia do Estado de São Paulo, SAESP. Os autores agradecem a revisão gramatical do texto original realizado pela Sra Joana Jacirene Costa Teixeira.

10. Referências

1. United Nations publication. UN commission reclassifies cannabis, yet still considered harmful [Internet]. UN News. [cited 2023 Sep 11]. Available from: https://news.un.org/en/story/2020/12/1079132

2. Häuser W, Finn DP, Kalso E, Krcevski-Skvarc N, Kress HG, Morlion B, et al. European Pain Federation (EFIC) position paper on appropriate use of cannabis-based medicines and medical cannabis for chronic pain management. Eur J Pain [Internet]. 2018 Oct;22(9):1547–64. Available from: https://onlinelibrary.wiley.com/doi/10.1002/ejp.1297

3. França C, Malunguinho E, Gama P, Helou M, Victor S, Freitas A, et al. Política estadual de fornecimento gratuito de medicamentos formulados de derivado vegetal à base de canabidiol, em associação com outras substâncias canabinoides, incluindo o tetrahidrocanabidiol [Internet]. Brazil: Assembléia Legislativa do Estado de São Paulo; 2023. Available from: https://www.al.sp.gov.br/repositorio/legislacao/lei/2023/lei-17618-31.01.2023.html

4. Eisenberg E, Ogintz M, Almog S. The Pharmacokinetics, Efficacy, Safety, and Ease of Use of a Novel Portable Metered-Dose Cannabis Inhaler in Patients With Chronic Neuropathic Pain: A Phase 1a Study. J Pain Palliat Care Pharmacother [Internet]. 2014 Sep 13;28(3):216–25. Available from: http://www.tandfonline.com/doi/full/10.3109/15360288.2014.941130

5. Aviram J, Samuelly-Leichtag G. Efficacy of Cannabis-Based Medicines for Pain Management: A Systematic Review and Meta-Analysis of Randomized Controlled Trials. Pain Physician [Internet]. 2017 Sep;20(6):E755–96. Available from: http://www.ncbi.nlm.nih.gov/pubmed/28934780

6. Gazendam A, Nucci N, Gouveia K, Abdel Khalik H, Rubinger L, Johal H. Cannabinoids in the Management of Acute Pain: A Systematic Review and Meta-analysis. Cannabis Cannabinoid Res [Internet]. 2020 Dec 1;5(4):290–7. Available from: https://www.liebertpub.com/doi/10.1089/can.2019.0079

7. Kopustinskiene DM, Masteikova R, Lazauskas R, Bernatoniene J. Cannabis sativa L. Bioactive Compounds and Their Protective Role in Oxidative Stress and Inflammation. Antioxidants [Internet]. 2022 Mar 29;11(4):660. Available from: https://www.mdpi.com/2076-3921/11/4/660

8. ANVISA - Agência Nacional de Vigilância Sanitária. Produtos canabinoides registrados na Agência Nacional de Vigilância Sanitária - Anvisa [Internet]. Produtos de Cannabis. [cited 2023 Sep 11]. Available from: https://consultas.anvisa.gov.br/#/cannabis/q/?situacaoRegistro=V

9. Finn DP, Haroutounian S, Hohmann AG, Krane E, Soliman N, Rice AS. Cannabinoids, the endocannabinoid system, and pain. Pain [Internet]. 2021 Mar 15;Publish Ah. Available from: https://journals.lww.com/10.1097/j.pain.0000000000002268

10. MacCallum CA, Russo EB. Practical considerations in medical cannabis administration and dosing. Eur J Intern Med [Internet]. 2018 Mar;49:12–9. Available from: https://linkinghub.elsevier.com/retrieve/pii/S0953620518300049

11. Wilson FR, Pan W, Schumsky DA. Recalculation of the Critical Values for Lawshe’s Content Validity Ratio. Meas Eval Couns Dev [Internet]. 2012 Jul 10;45(3):197–210. Available from: https://www.tandfonline.com/doi/full/10.1177/0748175612440286

12. Nasa P, Jain R, Juneja D. Delphi methodology in healthcare research: How to decide its appropriateness. World J Methodol [Internet]. 2021 Jul 20;11(4):116–29. Available from: https://www.wjgnet.com/2222-0682/full/v11/i4/116.htm

13. Maguire DR, France CP. Impact of Efficacy at the μ -Opioid Receptor on Antinociceptive Effects of Combinations of μ -Opioid Receptor Agonists and Cannabinoid Receptor Agonists. J Pharmacol Exp Ther [Internet]. 2014 Nov;351(2):383–9. Available from: http://jpet.aspetjournals.org/lookup/doi/10.1124/jpet.114.216648

14. Noyes R, Brunk SF, Avery DH, Canter A. The analgesic properties of delta-9-tetrahydrocannabinol and codeine. Clin Pharmacol Ther [Internet]. 1975 Jul;18(1):84–9. Available from: https://onlinelibrary.wiley.com/doi/10.1002/cpt197518184

15. Johnson JR, Burnell-Nugent M, Lossignol D, Ganae-Motan ED, Potts R, Fallon MT. Multicenter, Double-Blind, Randomized, Placebo-Controlled, Parallel-Group Study of the Efficacy, Safety, and Tolerability of THC:CBD Extract and THC Extract in Patients with Intractable Cancer-Related Pain. J Pain Symptom Manage [Internet]. 2010 Feb;39(2):167–79. Available from: https://linkinghub.elsevier.com/retrieve/pii/S0885392409007878

16. Lynch ME, Cesar-Rittenberg P, Hohmann AG. A Double-Blind, Placebo-Controlled, Crossover Pilot Trial With Extension Using an Oral Mucosal Cannabinoid Extract for Treatment of Chemotherapy-Induced Neuropathic Pain. J Pain Symptom Manage [Internet]. 2014 Jan;47(1):166–73. Available from: https://linkinghub.elsevier.com/retrieve/pii/S0885392413002388

17. Häuser W, Welsch P, Klose P, Radbruch L, Fitzcharles MA. Efficacy, tolerability and safety of cannabis-based medicines for cancer pain. Der Schmerz [Internet]. 2019 Oct 9;33(5):424–36. Available from: http://link.springer.com/10.1007/s00482-019-0373-3

18. Blake A, Wan BA, Malek L, DeAngelis C, Diaz P, Lao N, et al. A selective review of medical cannabis in cancer pain management. Ann Palliat Med [Internet]. 2017 Dec;6(S2):S215–22. Available from: http://apm.amegroups.com/article/view/16199/18209

19. To J, Davis M, Sbrana A, Alderman B, Hui D, Mukhopadhyay S, et al. MASCC guideline: cannabis for cancer-related pain and risk of harms and adverse events. Support Care Cancer [Internet]. 2023 Apr 6;31(4):202. Available from: https://link.springer.com/10.1007/s00520-023-07662-1

20. Dykukha I, Malessa R, Essner U, Überall MA. Nabiximols in Chronic Neuropathic Pain: A Meta-Analysis of Randomized Placebo-Controlled Trials. Pain Med [Internet]. 2021 Apr 20;22(4):861–74. Available from: https://academic.oup.com/painmedicine/article/22/4/861/6131796

21. Wallace MS, Marcotte TD, Atkinson JH, Padovano HT, Bonn-Miller M. A Secondary Analysis from a Randomized Trial on the Effect of Plasma Tetrahydrocannabinol Levels on Pain Reduction in Painful Diabetic Peripheral Neuropathy. J Pain [Internet]. 2020 Nov;21(11–12):1175–86. Available from: https://linkinghub.elsevier.com/retrieve/pii/S1526590020300146

22. Meng H, Johnston B, Englesakis M, Moulin DE, Bhatia A. Selective Cannabinoids for Chronic Neuropathic Pain. Anesth Analg [Internet]. 2017 Nov;125(5):1638–52. Available from: http://journals.lww.com/00000539-201711000-00032

23. Finnerup NB, Attal N, Haroutounian S, McNicol E, Baron R, Dworkin RH, et al. Pharmacotherapy for neuropathic pain in adults: a systematic review and meta-analysis. Lancet Neurol [Internet]. 2015 Feb;14(2):162–73. Available from: https://linkinghub.elsevier.com/retrieve/pii/S1474442214702510

24. Moulin D, Boulanger A, Clark A, Clarke H, Dao T, Finley G, et al. Pharmacological Management of Chronic Neuropathic Pain: Revised Consensus Statement from the Canadian Pain Society. Pain Res Manag [Internet]. 2014;19(6):328–35. Available from: http://www.hindawi.com/journals/prm/2014/754693/

25. Furrer D, Kröger E, Marcotte M, Jauvin N, Bélanger R, Ware M, et al. Cannabis against chronic musculoskeletal pain: a scoping review on users and their perceptions. J Cannabis Res [Internet]. 2021 Dec 4;3(1):41. Available from: https://jcannabisresearch.biomedcentral.com/articles/10.1186/s42238-021-00096-8

26. Huggins JP, Smart TS, Langman S, Taylor L, Young T. An efficient randomised, placebo-controlled clinical trial with the irreversible fatty acid amide hydrolase-1 inhibitor PF-04457845, which modulates endocannabinoids but fails to induce effective analgesia in patients with pain due to osteoarthritis of th. Pain [Internet]. 2012 Sep;153(9):1837–46. Available from: https://journals.lww.com/00006396-201209000-00013

27. Boehnke KF, Häuser W, Fitzcharles MA. Cannabidiol (CBD) in Rheumatic Diseases (Musculoskeletal Pain). Curr Rheumatol Rep [Internet]. 2022 Jul 3;24(7):238–46. Available from: https://link.springer.com/10.1007/s11926-022-01077-3

28. Khurshid H, Qureshi IA, Jahan N, Went TR, Sultan W, Sapkota A, et al. A Systematic Review of Fibromyalgia and Recent Advancements in Treatment: Is Medicinal Cannabis a New Hope? Cureus [Internet]. 2021 Aug 20; Available from: https://www.cureus.com/articles/65839-a-systematic-review-of-fibromyalgia-and-recent-advancements-in-treatment-is-medicinal-cannabis-a-new-hope

29. Walitt B, Klose P, Fitzcharles MA, Phillips T, Häuser W. Cannabinoids for fibromyalgia. Cochrane Database Syst Rev [Internet]. 2016 Jul 18; Available from: https://doi.wiley.com/10.1002/14651858.CD011694.pub2

30. van de Donk T, Niesters M, Kowal MA, Olofsen E, Dahan A, van Velzen M. An experimental randomized study on the analgesic effects of pharmaceutical-grade cannabis in chronic pain patients with fibromyalgia. Pain [Internet]. 2019 Apr 20;160(4):860–9. Available from: https://journals.lww.com/00006396-201904000-00011

31. Chaves C, Bittencourt PCT, Pelegrini A. Ingestion of a THC-Rich Cannabis Oil in People with Fibromyalgia: A Randomized, Double-Blind, Placebo-Controlled Clinical Trial. Pain Med [Internet]. 2020 Oct 1;21(10):2212–8. Available from: https://academic.oup.com/painmedicine/article/21/10/2212/5942556

32. Fitzcharles MA, Rampakakis E, Sampalis JS, Shir Y, Cohen M, Starr M, et al. Use of medical cannabis by patients with fibromyalgia in Canada after cannabis legalisation: a cross-sectional study. Clin Exp Rheumatol [Internet]. 2021 Jun 21;39(3):115–9. Available from: https://www.clinexprheumatol.org/abstract.asp?a=17212

33. Bourke SL, Schlag AK, O’Sullivan SE, Nutt DJ, Finn DP. Cannabinoids and the endocannabinoid system in fibromyalgia: A review of preclinical and clinical research. Pharmacol Ther [Internet]. 2022 Dec;240:108216. Available from: https://linkinghub.elsevier.com/retrieve/pii/S0163725822001103

34. Sagy I, Bar-Lev Schleider L, Abu-Shakra M, Novack V. Safety and Efficacy of Medical Cannabis in Fibromyalgia. J Clin Med [Internet]. 2019 Jun 5;8(6):807. Available from: https://www.mdpi.com/2077-0383/8/6/807

35. Abalo R, Martín-Fontelles MI. Cannabis, Cannabinoids, and Visceral Pain. In: Handbook of Cannabis and Related Pathologies [Internet]. Elsevier; 2017. p. 439–49. Available from: https://linkinghub.elsevier.com/retrieve/pii/B978012800756300051X

36. Picardo S, Kaplan GG, Sharkey KA, Seow CH. Insights into the role of cannabis in the management of inflammatory bowel disease. Therap Adv Gastroenterol [Internet]. 2019 Jan 3;12:175628481987097. Available from: http://journals.sagepub.com/doi/10.1177/1756284819870977

37. Bogale K, Raup-Konsavage W, Dalessio S, Vrana K, Coates MD. Cannabis and Cannabis Derivatives for Abdominal Pain Management in Inflammatory Bowel Disease. Med Cannabis Cannabinoids [Internet]. 2021 Jun 21;4(2):97–106. Available from: https://www.karger.com/Article/FullText/517425

38. Goyal H, Singla U, Gupta U, May E. Role of cannabis in digestive disorders. Eur J Gastroenterol Hepatol [Internet]. 2017 Feb;29(2):135–43. Available from: http://journals.lww.com/00042737-201702000-00002

39. Nickel JC. Medical marijuana for urologic chronic pelvic pain. Can Urol Assoc J [Internet]. 2018 Jun 11;12(6S3):S181-3. Available from: https://cuaj.ca/index.php/journal/article/view/5331

40. Carrubba AR, Ebbert JO, Spaulding AC, DeStephano D, DeStephano CC. Use of Cannabis for Self-Management of Chronic Pelvic Pain. J Women’s Heal [Internet]. 2021 Sep 1;30(9):1344–51. Available from: https://www.liebertpub.com/doi/10.1089/jwh.2020.8737

41. Tripp DA, Nickel JC, Katz L, Krsmanovic A, Ware MA, Santor D. A survey of cannabis (marijuana) use and self-reported benefit in men with chronic prostatitis/chronic pelvic pain syndrome. Can Urol Assoc J [Internet]. 2014 Dec 15;8(11–12):901. Available from: http://www.cuaj.ca/index.php/journal/article/view/2268

42. Sharkey KA, Wiley JW. The Role of the Endocannabinoid System in the Brain–Gut Axis. Gastroenterology [Internet]. 2016 Aug;151(2):252–66. Available from: https://linkinghub.elsevier.com/retrieve/pii/S0016508516343190

43. Okusanya BO, Lott BE, Ehiri J, McClelland J, Rosales C. Medical Cannabis for the Treatment of Migraine in Adults: A Review of the Evidence. Front Neurol [Internet]. 2022 May 30;13. Available from: https://www.frontiersin.org/articles/10.3389/fneur.2022.871187/full

44. Grossman S, Tan H, Gadiwalla Y. Cannabis and orofacial pain: a systematic review. Br J Oral Maxillofac Surg [Internet]. 2022 Jun;60(5):e677–90. Available from: https://linkinghub.elsevier.com/retrieve/pii/S0266435621002199

45. Votrubec C, Tran P, Lei A, Brunet Z, Bean L, Olsen B, et al. Cannabinoid therapeutics in orofacial pain management: a systematic review. Aust Dent J [Internet]. 2022 Dec 29;67(4):314–27. Available from: https://onlinelibrary.wiley.com/doi/10.1111/adj.12934

46. Abdallah FW, Hussain N, Weaver T, Brull R. Analgesic efficacy of cannabinoids for acute pain management after surgery: a systematic review and meta-analysis. Reg Anesth Pain Med [Internet]. 2020 Jul;45(7):509–19. Available from: https://rapm.bmj.com/lookup/doi/10.1136/rapm-2020-101340

47. Maione S, Costa B, Di Marzo V. Endocannabinoids: A unique opportunity to develop multitarget analgesics. Pain [Internet]. 2013 Dec;154(Supplement 1):S87–93. Available from: https://journals.lww.com/00006396-201312001-00010

48. Russo EB, Marcu J. Cannabis Pharmacology: The Usual Suspects and a Few Promising Leads. In 2017. p. 67–134. Available from: https://linkinghub.elsevier.com/retrieve/pii/S1054358917300273

49. Caspi A, Moffitt TE, Cannon M, McClay J, Murray R, Harrington H, et al. Moderation of the Effect of Adolescent-Onset Cannabis Use on Adult Psychosis by a Functional Polymorphism in the Catechol-O-Methyltransferase Gene: Longitudinal Evidence of a Gene X Environment Interaction. Biol Psychiatry [Internet]. 2005 May;57(10):1117–27. Available from: https://linkinghub.elsevier.com/retrieve/pii/S0006322305001034

50. Chesney E, Oliver D, Green A, Sovi S, Wilson J, Englund A, et al. Adverse effects of cannabidiol: a systematic review and meta-analysis of randomized clinical trials. Neuropsychopharmacology [Internet]. 2020 Oct;45(11):1799–806. Available from: http://www.ncbi.nlm.nih.gov/pubmed/32268347

51. Abramovici H. Information for Health Care Professionals: Cannabis (marihuana, marijuana) and the cannabinoids - Health Canada. Ethics [Internet]. 2013;9:152. Available from: https://www.canada.ca/en/health-canada/services/drugs-medication/cannabis/information-medical-practitioners/information-health-care-professionals-cannabis-cannabinoids.html

52. Dryburgh LM, Bolan NS, Grof CPL, Galettis P, Schneider J, Lucas CJ, et al. Cannabis contaminants: sources, distribution, human toxicity and pharmacologic effects. Br J Clin Pharmacol [Internet]. 2018 Nov;84(11):2468–76. Available from: http://www.ncbi.nlm.nih.gov/pubmed/29953631

53. United Nations. World Drug Report 2019. Booklet 5: CANNABIS AND HALLUCINOGENS [Internet]. Vienna, Austria; 2019. Available from: https://wdr.unodc.org/wdr2019/prelaunch/WDR19_Booklet_5_CANNABIS_HALLUCINOGENS.pdf

54. Taha T, Meiri D, Talhamy S, Wollner M, Peer A, Bar-Sela G. Cannabis Impacts Tumor Response Rate to Nivolumab in Patients with Advanced Malignancies. Oncologist [Internet]. 2019 Apr 1;24(4):549–54. Available from: https://academic.oup.com/oncolo/article/24/4/549/6439154

55. Côté M, Trudel M, Wang C, Fortin A. Improving Quality of Life With Nabilone During Radiotherapy Treatments for Head and Neck Cancers. Ann Otol Rhinol Laryngol [Internet]. 2016 Apr 25;125(4):317–24. Available from: http://journals.sagepub.com/doi/10.1177/0003489415612801

56. Zongo A, Lee C, Dyck JRB, El-Mourad J, Hyshka E, Hanlon JG, et al. Medical cannabis authorization and the risk of cardiovascular events: a longitudinal cohort study. BMC Cardiovasc Disord [Internet]. 2021 Sep 10;21(1):426. Available from: http://www.ncbi.nlm.nih.gov/pubmed/34507536

57. Fišar Z. Inhibition of monoamine oxidase activity by cannabinoids. Naunyn Schmiedebergs Arch Pharmacol [Internet]. 2010 Jun 18;381(6):563–72. Available from: http://link.springer.com/10.1007/s00210-010-0517-6

58. WILENS TE, BIEDERMAN J, SPENCER TJ. Case Study: Adverse Effects of Smoking Marijuana While Receiving Tricyclic Antidepressants. J Am Acad Child Adolesc Psychiatry [Internet]. 1997 Jan;36(1):45–8. Available from: https://linkinghub.elsevier.com/retrieve/pii/S0890856709636984

59. Schlag AK, Baldwin DS, Barnes M, Bazire S, Coathup R, Curran HV, et al. Medical cannabis in the UK: From principle to practice. J Psychopharmacol [Internet]. 2020 Sep 10;34(9):931–7. Available from: http://journals.sagepub.com/doi/10.1177/0269881120926677

60. Bhaskar A, Bell A, Boivin M, Briques W, Brown M, Clarke H, et al. Consensus recommendations on dosing and administration of medical cannabis to treat chronic pain: results of a modified Delphi process. J Cannabis Res [Internet]. 2021 Dec 2;3(1):22. Available from: https://jcannabisresearch.biomedcentral.com/articles/10.1186/s42238-021-00073-1

61. Jouanjus E, Raymond V, Lapeyre-Mestre M, Wolff V. What is the Current Knowledge About the Cardiovascular Risk for Users of Cannabis-Based Products? A Systematic Review. Curr Atheroscler Rep [Internet]. 2017 Jun 21;19(6):26. Available from: http://link.springer.com/10.1007/s11883-017-0663-0

62. Shah S, Schwenk ES, Sondekoppam R V, Clarke H, Zakowski M, Rzasa-Lynn RS, et al. ASRA Pain Medicine consensus guidelines on the management of the perioperative patient on cannabis and cannabinoids. Reg Anesth Pain Med [Internet]. 2023 Mar;48(3):97–117. Available from: http://www.ncbi.nlm.nih.gov/pubmed/36596580

63. Hutchings DE, Martin BR, Gamagaris Z, Miller N, Fico T. Plasma concentrations of delta-9-tetrahydrocannabinol in dams and fetuses following acute or multiple prenatal dosing in rats. Life Sci [Internet]. 1989 Jan;44(11):697–701. Available from: https://linkinghub.elsevier.com/retrieve/pii/0024320589903809

64. Bertrand KA, Hanan NJ, Honerkamp-Smith G, Best BM, Chambers CD. Marijuana Use by Breastfeeding Mothers and Cannabinoid Concentrations in Breast Milk. Pediatrics [Internet]. 2018 Sep 1;142(3). Available from: https://publications.aap.org/pediatrics/article/142/3/e20181076/81671/Marijuana-Use-by-Breastfeeding-Mothers-and

65. Vaessen TSJ, de Jong L, Schäfer AT, Damen T, Uittenboogaard A, Krolinski P, et al. The interaction between cannabis use and the Val158Met polymorphism of the COMT gene in psychosis: A transdiagnostic meta - analysis. PLoS One [Internet]. 2018;13(2):e0192658. Available from: http://www.ncbi.nlm.nih.gov/pubmed/29444152
