# Supplementary material for: Cannabinoid products for pain management: recommendations from the São Paulo State Society of Anesthesiology
Source: Braz J Anesthesiol. 2024 May 11;74(4):844513. doi: 10.1016/j.bjane.2024.844513 (PMC11167254; doi:10.1016/j.bjane.2024.844513)
Supplement: Supplementary file 2 [file mmc2.docx]

| Fármaco | Efeito da medicação | Classe de Canabinóide | Canabinoide | Inibidor de enzima | Indutor de enzima |
| --- | --- | --- | --- | --- | --- |
| acenocumarol | ↓↑ | CBD | Canabidiol | ✓ | ✓ |
|  | ↑ | THC | Dronabinol ou Nabilona | ✓ (fraco) |  |
|  | ↓↑ | THC/CBD | Nabiximols | ✓ | ✓ |
| alfentanil | ↑ | THC | Dronabinol ou Nabilona | ✓ (fraco) |  |
|  | ↓↑ | THC/CBD | Nabiximols | ✓ | ✓ |
| alosetron | ↓↑ | CBD | Canabidiol | ✓ | ✓ |
|  | ↓ | THC/CBD | Nabiximols |  | ✓ |
| alprazolam | ↑ | THC | Dronabinol ou Nabilona | ✓ (fraco) |  |
|  | ↓↑ | THC/CBD | Nabiximols | ✓ | ✓ |
| aminofilina | ↓↑ | CBD | Canabidiol | ✓ | ✓ |
|  | ↑ | THC | Dronabinol ou Nabilona | ✓ (fraco) |  |
|  | ↓↑ | THC/CBD | Nabiximols | ✓ | ✓ |
| amiodarona | ↓↑ | CBD | Canabidiol | ✓ | ✓ |
|  | ↑ | THC | Dronabinol | ✓ (fraco) |  |
|  | ↑ | THC | Nabilona | ✓(moderado) |  |
|  | ↓↑ | THC/CBD | Nabiximols | ✓ | ✓ |
| amitriptilina | ↓↑ | CBD | Canabidiol | ✓ | ✓ |
|  | ↑ | THC | Dronabinol ou Nabilona | ✓ (fraca) |  |
|  | ↓↑ | THC/CBD | Nabiximols | ✓ | ✓ |
| anfotericina B | ↑ | THC | Dronabinol |  |  |
| aprepitant | ↑ | THC | Dronabinol ou Nabilona | ✓ (fraca) |  |
|  | ↓↑ | THC/CBD | Nabiximols | ✓ | ✓ |
| argatroban | ↑ | THC | Dronabinol ou Nabilona | ✓ (fraca) |  |
|  | ↓↑ | THC/CBD | Nabiximols | ✓ | ✓ |
| astemizol | ↑ | THC | Dronabinol e Nabilona | ✓ (fraca) |  |
|  | ↓↑ | THC/CBD | Nabiximols | ✓ | ✓ |
| atorvastatina | ↑ | THC | Dronabinol e Nabilona | ✓ (fraca) |  |
|  | ↓↑ | THC/CBD | Nabiximols | ✓ | ✓ |
| avanafil | ↑ | THC | Dronabinol e Nabilona | ✓ (fraca) |  |
|  | ↓↑ | THC/CBD | Nabiximols | ✓ | ✓ |
| bromocriptina | ↑ | THC | Dronabinol e Nabilona | ✓ (fraca) |  |
|  | ↓↑ | THC/CBD | Nabiximols | ✓ | ✓ |
| budesonida | ↑ | THC | Dronabinol e Nabilona | ✓ (fraca) |  |
|  | ↓↑ | THC/CBD | Nabiximols | ✓ | ✓ |
| bupropiona | ↑ | CBD | Canabidiol | ✓ | ✓ |
| buspirona | ↑ | THC | Dronabinol e Nabilona | ✓ (fraco) |  |
|  | ↓↑ | THC/CBD | Nabiximols | ✓ | ✓ |
| busulfan | ↑ | THC | Dronabinol e Nabilona | ✓ (fraco) |  |
|  | ↓↑ | THC/CBD | Nabiximols | ✓ | ✓ |
| cafeina | ↓↑ | CBD | Canabidiol | ✓ | ✓ |
|  | ↓ | THC/CBD | Nabiximols |  | ✓ |
| carbamazepina | ↓↑ | CBD | Canabidiol | ✓ | ✓ |
|  | ↑ | THC | Dronabinol e Nabilona | ✓ (fraco) |  |

Quadro suplementar: Interações medicamentosas das substâncias canabinoides.
